# Time to plan for Plan S

**DOI:** 10.1002/nop2.263

**Published:** 2019-03-15

**Authors:** Roger Watson, Mark Hayter

Heard of “Plan S”? You will. Plan S arose from the work of an international group called Coalition S. Their aim is to have all published research available open access immediately on publication. The coalition has some powerful membership organizations, mainly across Europe but in some other countries too. Coverage is not yet universal, and some key organizations have not signed up. However, the coalition has one powerful financial backer in the Bill & Melinda Gates Foundation and, given the widespread—and sometimes misplaced—enthusiasm for open access, this is likely to gather momentum. On the face of it “Plan S” seems entirely laudable and altruistic, however, it raises a number of issues for both researchers and publishers.

## 1 RESEARCHERS

Researchers will have to obtain funding to publish open access if their funding body is a member of Coalition S. Open access is not cheap with some journals charging several thousand pounds in APCs (article processing charges). Few individuals will be prepared to pay from their own pockets or to have funding available for this. Only a few universities will have funding to spare, although more will have subscriptions to PLOSOne (Public Library of Science) and BMC (Biomed Central) whereby their staff may submit, without the need to pay an APC, to journals published by these two major open access publishing houses. An alternative source of funding is via the research funding bodies, if they decide to make such funding available, and it seems likely that they will. But these are publicly funded bodies who will either require more money from their governments to accommodate this or will have to divert resources from research discovery funding to open access funding. Either way, this has consequences for those who ultimately fund such research: the general tax‐paying public who will either—without consultation—have a deficit in research, a deficit in other government‐funded amenities or an increase in tax. Open access, but hardly democracy.

## 2 PUBLISHERS

One premise of Plan S is that researchers may no longer publish in “hybrid” journals: those that pre‐existed open access publishing, which perpetuate the “pay to view” model but which now offer an open access option by APC. This leaves very few reputable journals *per se* where researchers may publish. But if they want to publish in reputable journals that are also Clarivate listed with an impact factor then the field becomes very narrow. The consequences for publishers vary from carrying on regardless and continuing to publish articles not emanating from research funded by Coalition S organizations to going out of business (Pells, 2019). However, in‐between, there are options such as moving hybrid journals to being completely open access and developing more open access journals.

## 3 DANGERS AND OPPORTUNITIES

Apart from the danger to publishers and some well‐established journals, there is the danger that the insistence on open access publishing will create further opportunities for low quality and predatory open access publishers. There are at least 10,000 verified predatory journals (Watson, 2019), and the distinction between those which are genuine predators and those which are merely low quality—both options being undesirable to reputable researchers—is very blurred. Nevertheless, the temptation to publish in low quality and predatory publishers may be too great for some researchers and, currently, despite calls for action (Watson, 2017a) there remains no open access lists of journals considered to be either reputable or predatory. Jeffrey Beall did his best, but he was working alone and eventually silenced by his employers (Watson, 2017b). Since then, Cabell's International provides a list but it costs tens of thousands of dollars to gain access.

Aside from the dangers, there are also opportunities. There are a growing number of legitimate open access journals from all the major publishing houses and these may see an increase in submission with a concomitant increase in income. This may save the publishers from financial peril. However, if Coalition S grows and becomes more influential, it is conceivable that they will try to drive down the cost of open access publishing, specifically through lower APCs. This will not be good for publishers. Their profits are frequently described as being “excessive”—without a definition of “excessive” being offered (Watson, 2016). But all the major publishing houses manage journals that make no profit, and this is enabled by the fact that they run a small number of very profitable journals. Publishers are not charities, they are businesses, but Coalition S and others in the academic community who make calls for greater open access need to realize that they may no longer have the luxury of such a wide range of reputable journals in which to publish.

Another open access option exists and that is diamond open access. Currently, very few journals operate this model and one of these is the *WikiJournal of Medicine*. This journal—and others in the Wikiversity suite—are funded by the WikiMedia Foundation (which also funds Wikipedia) and this is, essentially, “crowdfunded.” As such it is an attractive model but once you become involved in submitting to and editing one of these journals you realize what the major publishing houses have to offer. The main thing they offer is online journal managing platforms and full‐time staff to support editorial teams. But it is possible that this model could see an injection of cash from some philanthropist who supports open access.

## 4 COALITION S

Of course, Coalition S might attenuate its plans and it will be another case of *plus ça change*. Whatever Plan S achieves for Coalition S in terms of increasing open access and forcing the hands of publishers, they should recognize how we got where we are, with a plethora of reputable journals in which to publish and “Rolls Royce” journal management systems. This was not funded by open access and, unless open access delivers income to the publishers, we endanger these systems.
